# Ingestive Behavior of Young Lambs on Contrasting Tropical Grass Sward Heights

**DOI:** 10.3389/fvets.2020.00643

**Published:** 2020-09-17

**Authors:** Joseane Anjos da Silva, Cesar Henrique Espirito Candal Poli, Jalise Fabíola Tontini, Lívia Raymundo Irigoyen, Elisa Cristina Modesto, Juan Jose Villalba

**Affiliations:** ^1^Departamento de Zootecnia, Universidade Federal do Rio Grande do Sul, Porto Alegre, Brazil; ^2^Department of Wildland Resources, Utah State University, Logan, UT, United States

**Keywords:** decision tree, grazing time, Capim Aruana, height, biting rate

## Abstract

The efficiency of grazing ruminant production systems is directly associated to the animals' ingestive behavior, and to structural characteristics of the pastures. The objective of this study was to evaluate the ingestive behavior of young lambs grazing three different heights of Capim Aruana (*Panicum maximum*). The experiment was carried out in two consecutive years, in which 30 tester lambs (4–5 months old) were equally divided into three paddocks (treatments) corresponding to different average sward heights of Aruana grass: (1) Tall-75 cm; (2) Medium-50 cm; and (3) Short-25 cm in a randomized block design. Ingestive behavior assessments were carried out every 28 days through 10-min observations of the main activities of the animals (grazing, ruminating, idling) and biting rate, from sunrise to sunset. In addition, the productive and qualitative characteristics of the pastures were assessed. Despite differences in pasture structure, grazing time (GT) and idling time were similar among treatments (*P* = 0.4266 and *P* = 0.2939, respectively). The shortest ruminating time (RT, *P* = 0.0181) was recorded in the treatment of lowest sward height. Lambs grazing on this treatment also showed 23% more bites per minute (*P*= < 0.0001) than animals in the Tall and Medium treatments. A Decision Tree analysis was performed for GT, identifying in a hierarchical order that the initial weight of the animals and sward height explained 62% (*R*^2^ = 0.621) of the variation, representing the variables with the greatest influence on GT. Initial body weight explained 48% of the model. Thus, our research shows that the different sward heights of Capim Aruana mainly alter the lamb's RT and biting rate, and that the animals' initial body weight is a key factor influencing GT, given that this variable makes lambs more susceptible to changes in sward height.

## Introduction

Pasture production systems represent a significant opportunity for increasing the sustainable production of ruminant animals worldwide. Under this scenario, animal performance depends of sward attributes such the quality and quantity of forage harvested during the grazing process (1). For instance, the ingestive behavior of young lambs kept on tropical pastures may be influenced by the different structures of swards at which they are exposed during grazing.

Sheep are highly selective animals, a trait that differentiates them from other, larger herbivores (2). This characteristic of selectivity inherent to the species is even more important in young lambs, as they progress through complex feeding periods and behavioral transitions. In addition, lambs are positioned at an optimal point on their growth curve, as they display high levels of intake and performance rates during this period (3). Because of these specific characteristics, it is necessary to understand the ingestive behavior of animals during this critical period, as it is one of the aspects that determines performance. In animal production systems, feeding is one of the most limiting factors for obtaining good results in productive performance (4). The way in which the forage is available to the animal is known as forage structure, which is responsible for the amount of nutrients ingested during the grazing process (5). According to Silva et al. (6) the structural characteristics of forage plants directly interfere with the ingestive behavior and performance of grazing animals, which, in turn alter the morphological (height, mass, and density) and physiological (photosynthetic rate and phenological stage) characteristics of the forage canopy, modifying subsequent animal and plant responses to grazing.

Tropical grasses are characterized by their high structure and growth rate (7, 8). These characteristics become a relevant issue when we think about the use of these forages for lamb production. Lambs are relatively small animals and seize food with their lips. In tall pastures with leaves above the animals' heads, lambs need to harvest practically leaf by leaf during the grazing process (5). The maintenance of the pasture structure is an important point to be analyzed when only this category is used in the pastoral system, since the growth rate of these pastures may be greater than the pasture harvesting capacity of young small ruminants. Consequently, there will be an accumulation of the most fibrous components of the sward with decreased nutritional quality of the forage on offer. For these reasons, proper management of the structure (height) is important to allow the best use of tropical grasses by young weaned lambs.

The ingestive behavior of grazers in temperate pastures is already well-known and described as the linear relationship between the decrease in height of the forage canopy and the increase in grazing time (9). Nevertheless, knowledge on such interaction is still scarce for tropical forage species and small ruminants. In order to obtain greater efficiency in the production systems of grazing ruminants, it is essential to know the animals' ingestive behavior and its relationships with forage structure. In addition to forage structure, it is necessary to understand how the nutritional composition of forages interact with their structural characteristics to influence foraging behavior and animal performance. Therefore, the objective of this study was to evaluate the ingestive behavior of young lambs grazing Capim Aruana (*Panicum maximum* cv. IZ-5) of different structures.

## Materials and Methods

### Experimental Proceedings

The experiment was conducted during two consecutive years at the Experimental Agronomic Station of the Universidade Federal do Rio Grande do Sul, located at 46, Eldorado do Sul, Brazil—Latitude 29° 13 '26 “S, Longitude 53° 40 '45” W. The climate is subtropical humid “cfa” according to the Köppen (10) classification. The cfa classification is characterized by hot summers with temperature averages over 22°C in the hottest month and well distributed rains (11). The experiment was carried out for 56 days during the summer in the years 2018 and 2019 (between January and March). Before this experimental period, an adaptation period of 7 days was performed to familiarize animals to their environment.

The treatments were characterized by different aimed structures of Capim Aruana (*Panicum maximum*), represented by different pasture heights: (1) Tall Treatment −75 cm of average height; (2) Medium Treatment −50 cm of average height; and (3) Short Treatment −25 cm of average height. To maintain the different pasture structures, strategic mowing was carried out before each experimental period. The pasture was mowed at 5 cm of residual height in all experimental paddocks, performed at different times before the beginning of the experiment (Tall −4 weeks; Medium −2 weeks; Short −1 week before the beginning of the experiment).

For experimental evaluations, 30 young weaned tester lambs with an average age of 4–5 months (at the beginning of the experiment), were used in each year of the study. Lambs were randomly distributed across groups and pastures, considering the variation of gender (female and castrated male) and weight, resulting in a uniform distribution of animals within each treatment group (*N* = 10 lambs/group). A continuous grazing method was used and all treatments had a 12% herbage allowance [12 kg total dry matter (DM) per 100 kg of animal bodyweight (BW)/day] adjusted in the day 1 of the experiment and every 28 days using the “put and take” technique (12). According to this technique, there were two groups of animals, one called “testers” that grazed continuously and showed the effect of the treatments, and another group name “put-and-take” lambs used only to maintain the sward height and regulate forage allowance. The lambs had access to shade, and water and mineral salt in *ad libitum* amounts. The average initial weight of the animals was similar between treatments (21 kg, *P* = 0.9401). Lambs were weighed every 28 days with a previous 12 h fasting of solids and liquids.

### Pasture Assessments

Sward height was checked every 7 days using random sampling, using a 1.5-m sward stick (13), taking measurements on 52 random points for each paddock, measuring the highest point of the leaf from the ground. The forage structure is composed not only by height, but also by density, forage mass and plant stage. Height, however, is a measure of high correlation with the forage structure and easy to measure, allowing for a high number of measurements during weekly intervals.

Evaluations to estimate herbage mass were carried out on day 1 of the experiment and every 28 days thereafter using a 0.25 m^2^ frame, totaling six sample points per treatment, three at the average pasture height and three at random. These samples were cut close to the ground, collected and weighed. The samples were homogenized, and two sub-samples were taken, one for determining the percentage of dry matter (DM), and another for botanical separation in leaf blade, stem + sheath, inflorescence, other grasses, other legumes, other species and senescent material. The separation of the plant's morphological components allows for the calculation of the leaf: stem ratio, which was the main variable that characterized the pasture. After botanical separation, all subsamples were placed in a forced air oven at an average temperature of 60°C until constant weight, when samples were weighed on a 0.1-g precision balance.

The daily forage accumulation rate was measured every 28 days, using three grazing exclusion cages per paddock, according to Kinglmann et al. (14). The objective of the evaluation was to measure the daily rate of pasture growth, enabling subsequent calculations of forage supply and adjustment of stocking rate. The daily forage accumulation was estimated by the difference between the sample cut inside the cage in the present period, and the forage mass cut in the previous period outside the cage, divided by the number of days in the period.

Forage samples were collected every 28 days using the grazing simulation technique (15) to assess the nutritional quality of forages. Bromatological analyses of forage samples were made to estimate the contents of dry matter (DM, method n° 930.15), mineral matter (MM, method n° 942.05) and crude protein (CP, method n° 984.13), according to the AOAC methodology (16). The analysis of apparent *in vitro* digestibility of organic matter (DIVMO) was performed according to Tilley and Terry (17). The neutral detergent fiber (NDF) concentration was analyzed according to Van Soest et al. (18), while acid detergent fiber (ADF) and acid detergent lignin (ADL) according to Goering and Van Soest (19). Determinations of insoluble nitrogen in neutral detergent (NIND) and insoluble nitrogen in acid detergent (NIAD) were also carried out according to the methodology described by Licitra et al. (20).

### Ingestive Behavior Evaluation

The assessment of ingestive behavior was performed with continuous notes during the day (from sunrise to sunset) every 10 min by trained people using the method described by Jamieson and Hodgson (21). The observations were performed only during daytime because most of the grazing activities of ruminants occur during this period (22–25) and nocturnal observations were not possible due to the difficulties to visualize the animals in tall pastures at night. In addition, the effect of sward height could certainly be visualized during the day. The animals were individually identified with fabric collars, in which each animal in the paddock received a collar with a different color. The activities of grazing, ruminating and idling were recorded individually for each tester animal. These assessments were carried out every 28 days. The temperature and relative humidity of the air were also measured.

The ruminating time (RT) was considered the period when the animal was not grazing but when it was chewing the ruminal bolus. The idling time (IT) represented the period when the animal was neither grazing nor ruminating. Grazing time (GT) was the period where the animal was actively grazing or selecting forage, including the period used for displacement during selection of the diet. Within the 10 min of GT assessments, the biting rate was recorded using the “20 bites method” described by Forbes et al. (26), which counts the time spent by the animal to take 20 bites.

### Meteorological Data From the Trial Period

In the first year of the experiment (2018), the average temperature was 23.2°C, 75.6% average relative humidity and 106.1 millimeters of rain during the experimental period. In the second year of the experiment (2019), the average temperature was 24.1°C, 74.8% relative humidity and 47.9 millimeters of rain. In the first behavioral assessment in 2018, the daily average temperature was 22.7°C and in the second, 19.7°C. In the year 2019, the average daily temperature in the first assessment was 25.5°C and in the second 26.3°C.

### Statistical Analysis

The experimental design used was randomized blocks, in which each year represented a block. Animals were considered the experimental units for variables related to ingestive behavior, and paddocks were considered the experimental units for pasture variables. Analysis of variance were performed to determine the effects of the treatments using the Mixed procedure in SAS 9.4, and the means were compared by the Tukey test at the 5% significance level. The variables evaluated over time, within each year, were considered as repeated measures. In addition to the analysis of variance, correlation analysis between the animal behavior and pasture variables were performed.

The ANOVA model included as fixed effects block, treatment, period (repeated measures over time within each year) and treatment x period interaction. The data of total GT and total IT were not normal (Shapiro-Wilk; *P* ≤ 0.05) and were transformed by log and square root, respectively. The results are presented as means adjusted by the LSMEANS (least square means) procedure of SAS (version 9.4, SAS Institute Inc., Cary, NC, USA), ± standard error of the mean. The LSMEANS procedure was used because least square means are less sensitive to missing data (27).

The data were also submitted to multivariate Decision Tree analysis performed by JMP software (version 12, SAS Institute Inc., Cary, NC, USA). This analysis allows to understand a result obtained by investigating the degree of interference that the factors studied may have in a given process of interest. The statistical program generates an equation that explains (through *R*^2^ value) which factors most influence a certain variable like GT. The independent variables included as factors in the analysis were initial body weight, herbage mass (DM/ha), leaf:stem ratio, pasture accumulation rate, leaf/ha, stem/ha, senescence/ha, inflorescence/ha, sward height and biting rate.

## Results

### Ingestive Behavior

The different structures of the Capim Aruana tropical pasture did not influence GT or IT by young lambs (*P* = 0.4266 and *P* = 0.2939, respectively), with averages for GT of 391.1 ± 15.44 min in the Tall; 389.1 ± 12.3 min in the Medium and 428.1 ± 24.3 min per day in the Short treatment. For the variable IT (*P*= 0.2939) the averages were 174.3 ± 25.8 min in the Tall; 152.9 ± 16.8 min in the Medium and 144.1 ± 21.7 min per day in the Short treatment. In relation to RT there was a significant (*P*= 0.0181) difference between treatments, being the longest times for the Medium treatment (174.4 ± 8.6 min/ day), which differed from the Short treatment (143.5 ± 7.2 min/ day). The RT in the Tall treatment (153.0 ± 7.3 min/day) did not differ from the other treatments (*P* > 0.05).

There was an interaction between treatment and period (*P* = 0.0049) in relation to biting rate, in which the Short treatment had similar biting rates in both periods, 1 and 2 (30.0 ± 1.4 and 32.6 ± 1.2 bits/min, respectively). Animals under the Medium and Tall treatments showed lower biting rates during both periods, as shown in Table 1.

**Table 1:** Ingestive behavior of lambs recently weaned in different structures of tropical pasture Capim Aruana (*Panicum maximum)* a with period 1 being the initial instant of forage offered to the animals (summer) and period 2 the end of the pasture cycle (next to autumn).

**Variables^a^**	**Period**	**Treatments**			***P-*****value**^b^		
		**Tall**	**Medium**	**Short**	**Treat**	**Per**	Treat*Per
GT (min)	1	383.4 ± 17.5	383.7 ± 13.4	409.3 ± 38.4	0.4266	0.0941	0.2541
	2	399.4 ± 26.2	394.2 ± 20.7	446.8 ± 30.9			
	Mean	391.1 ± 15.44	389.1 ± 12.3	428.1 ± 24.3			
IT (min)	1	205.8 ± 19.4	172.0 ± 16.4	173.9 ± 36.5	0.2939	0.0003	0.4540
	2	142.9 ± 26.0	133.8 ± 18.0	114.3 ± 22.5			
	Mean	174.3 ± 25.8	152.9 ± 16.8	144.1 ± 21.7			
RT (min)	1	130.3 ± 8.2	160.0 ± 12.8	132.7 ± 9.1	0.0181	<0.0001	0.2995
	2	177.0 ± 9.3	188.0 ± 11.1	154.3 ± 10.9			
	Mean	153.0 ± 7.3AB	174.4 ± 8.6A	143.5 ± 7.2B			
Bite Rate (bites/min)	1	19.8 ± 0.8c	25.6 ± 1.0b	30.0 ± 1.4ab	<0.0001	0.0006	0.0049
	2	26.8 ± 1.5b	25.6 ± 1.0b	32.6 ± 1.2a			
	Mean	23.2 ± 1.0B	25.6 ± 0.7B	31.3 ± 0.8A			

a *Variables = GT, grazing time; IT, Idling time; RT, ruminating time*.

b Different capital letters differ on the line for each variable; Different lowercase letters differ from each other in the treatment *period interaction for each variable analyzed.

**Figure 1:**
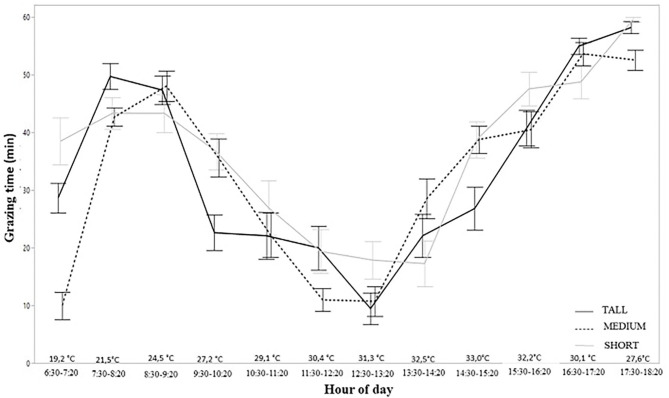
Behavior of grazing activity during the day of freshly weaned lambs in different structures of tropical pasture Capim Aruana (*Panicum maximum*).

When analyzing the GT of the animals over the different hours of the day, a similar pattern of behavior was observed in all treatments. In general, two grazing peaks occurred throughout the day. There was a peak in the morning with an average duration of 2 h between 07:30 am and close to 09:20 am. During this interval animals grazed more than 80% of the activities recorded. The animals returned to grazing activities after the hottest times of the day, around 02:20 pm, and the amount of time they spent grazing gradually increased, reaching almost 100% of the activities recorded after 05:20 pm (Figure 1).

Although the patterns of behavior were similar among treatments, it was possible to identify a different behavior of the Short treatment animals. While at the beginning of the behavior evaluation, at 6:30 am, the animals of this treatment were already in high grazing activity, almost 70% of the time, the animals on the Medium and Tall treatments were slowly starting their grazing activities. This behavior change shows that animals in the Low treatment started their grazing activities earlier in the day than in the other treatments, as shown in Figure 1. In addition to this behavior, animals under the Short treatment started to gradually reduce their grazing activity around 9:30 am, while this decline occurred for the other treatments at the same time but in a more pronounced manner. Another difference in behavior for animals in the Short treatment was that most of them did not cease their grazing activity in the hottest hours (11:30 am−02:20 pm), in contrast to animals assigned to taller structures (Figure 1).

**Figure 2:**
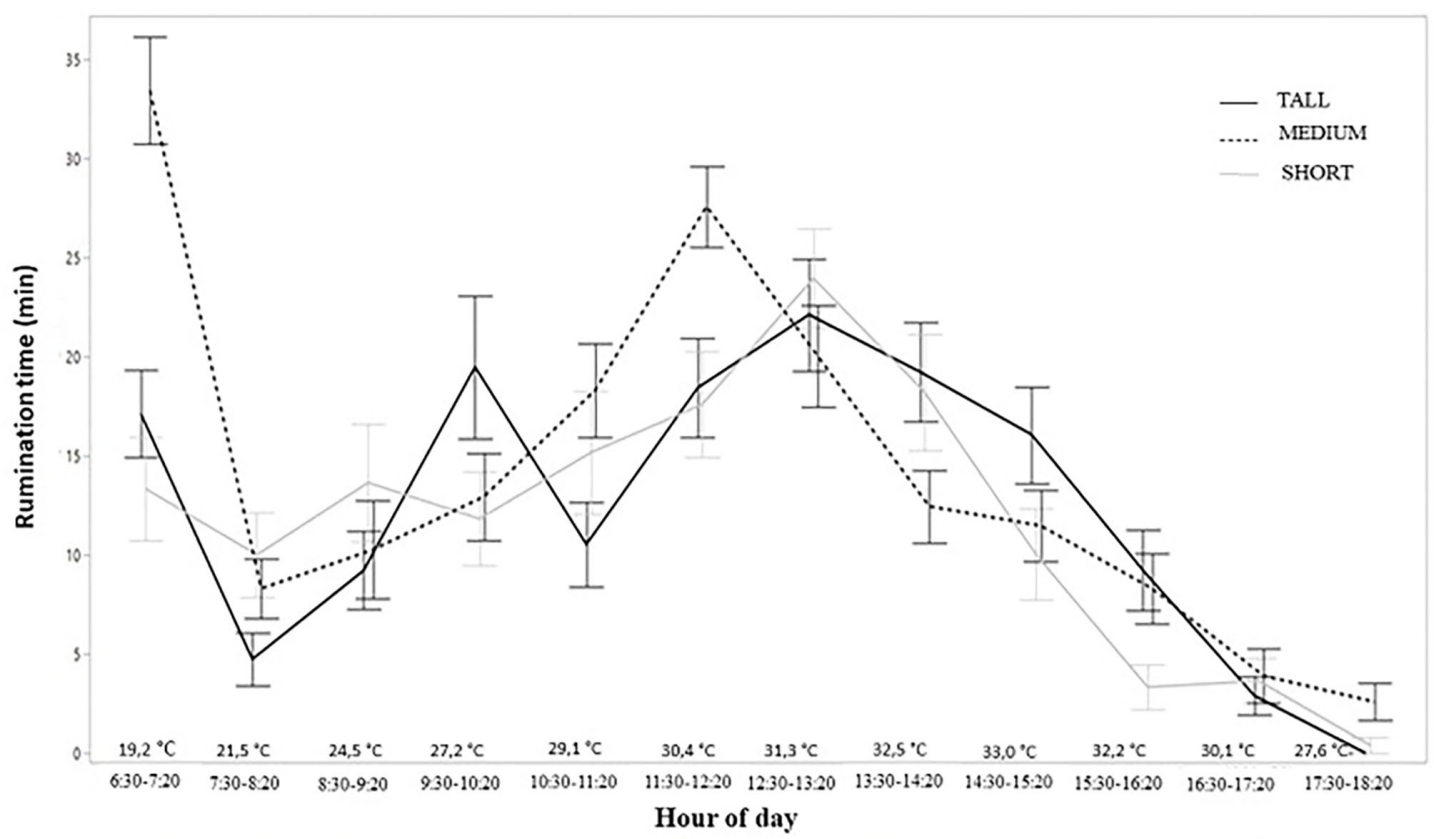
Rumination behavior activity during the day of freshly weaned lambs in different structures of tropical pasture Capim Aruana (*Panicum maximum*).

There was a small difference between the Medium and the other treatments with regards to RT. During the first hour, the percentage of time spent ruminating by animals in the Medium treatment was greater than for the other two treatments, and greater rumination activity was observed by these animals in the hottest periods of the day (Figure 2).

**Figure 3:**
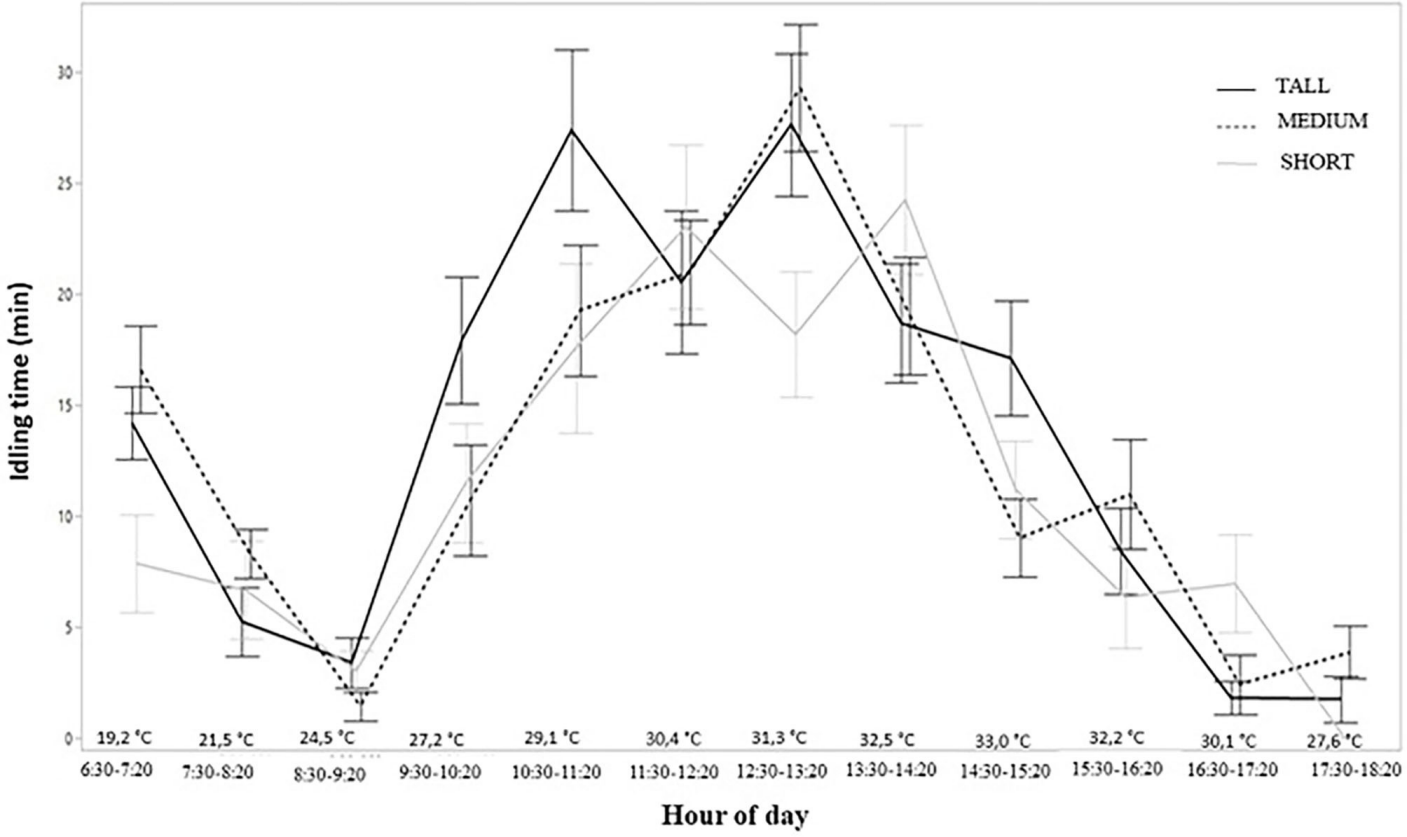
Idling behavior activity during the day of freshly weaned lambs in different structures of tropical pasture Capim Aruana (*Panicum maximum*).

Idling behavior across different hours of the day was similar for all treatments. In contrast to GT, idling behavior was less in the early morning and late afternoon, and there was an idling peak between 11:20 am 02:20 pm (Figure 3).

### Decision Tree Analysis

**Figure 4:**
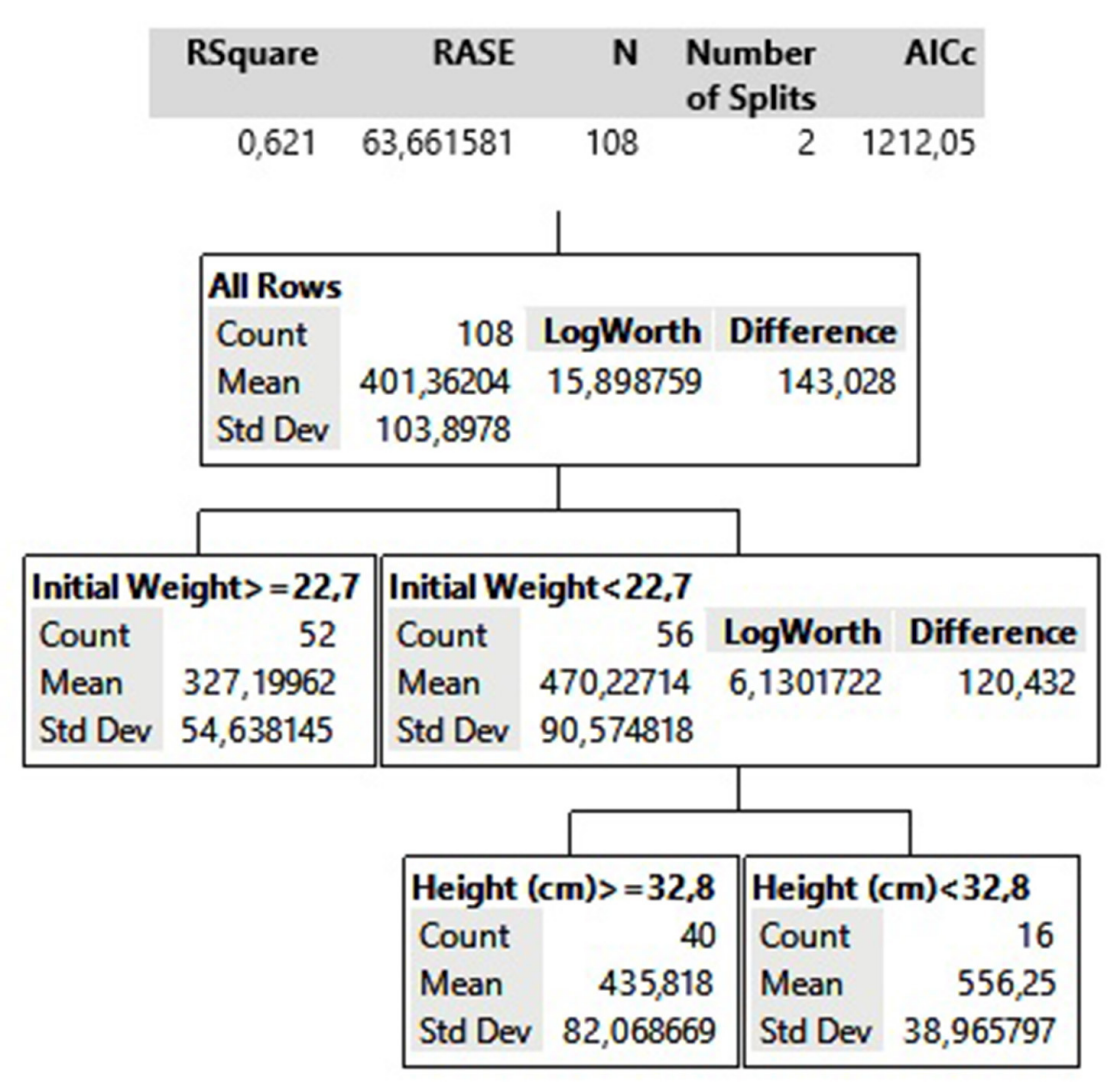
Decision Tree model for grazing time behavior variable.

According to this analysis, it was hierarchically identified that the initial weight of the animals and pasture height explained 62% (*R*^2^ = 0.621) of the model, being the variables of greatest influence in the animals GT. The first division of the model showed that the factor of greatest interference in GT was the initial weight of the animals (Figure 4), explaining 48% of the variation in the model.

The model estimated that animals with an average initial weight >22.7 kg would have an average GT of 327.1 min and animals weighing <22.7 kg would have an average GT of 470.2 min. For animals of lower weight, the analysis showed that the factor with the greatest influence at explaining the observed variability in GT was forage height. This result demonstrates that the pasture height (structure) is more important for smaller animals.

### Pasture

The pasture height showed a negative correlation with biting rate (*r* = −0.46, *P* < 0.0001). Another characteristic of the pasture that showed a significant relationship with biting rate was the number of inflorescences per hectare (*r* = −0.51, *P* < 0.0001). There was a greater amount of inflorescence in the taller swards at the end of the experiment. On average, there was a trend (*P* = 0.0967) for greater amount of inflorescences in the Tall treatment. In fact, a high correlation was observed between sward height and percentage of inflorescence (*r* = 0.81, *P* < −0.0001).

The variables leaf/ha and dead matter/ha showed no significant difference between treatments. The variable herbage mass (DM/ha) differed significantly (*P* < 0.0001) between treatments, being greater for the Tall treatment (Table 2). The pasture accumulation rate showed a trend (*P* = 0.0513) for greater values in the Tall than in the Short treatment, and significant correlations with ingestive behavior variables, like IT (*r* = 0.63, *P* < 0.0001) and GT (*r* = −0.49, *P* < 0.0001).

**Table 2:** Grazing variables in different structures of Capim Aruana (*Panicum maximum)* with period 1 being the initial instant of forage offered to the animals (summer) and period 2 the end of the pasture cycle (near autumn).

**Variables^a^**	**Period**	**Treatments**			***P-*****value^b^**		
		**Tall**	**Medium**	**Short**	**Treat**	**Per**	Treat*per
DM/ha (Kg)	1	4167.8 ± 111.1	2681.7 ± 129.6	1639 ± 37	<0.0001	0.0010	0.8141
	2	5513 ± 143.18	3820.8 ± 85.3	2937 ± 51.4			
	Mean	4840.1 ± 144.7A	3251.3 ± 117.4B	2288 ± 120.6B			
Accumulation Rate (Kg/day)	1	195.1 ± 8.7	104.8 ± 5.3	127.1 ± 20.5	0.0513		
	2	−9.8 ± 0.2	4.8 ± 15.1	32.2 ± 22.2			
	Mean	92.6 ± 17.8	54.8 ± 11.1	79.6 ± 17.1			
Height (cm)	1	79.9 ± 1.2a	44.4 ± 2.7bc	23.8 ± 0.9d	<0.0001	0.7735	0.0120
	2	67.2 ± 0.7ab	50.9 ± 0.4b	25.3 ± 0.6cd			
	Mean	73.5 ± 1.2A	47.7 ± 1.4B	24.5 ± 0.5C			
L:S	1	1.4 ± 0.1	1.4 ± 0	3.7 ± 0.3	0.0006	0.6946	0.2512
	2	0.3 ± 0	1.4 ± 0.1	1.4 ± 0.1			
	Mean	0.8 ± 0.1B	1.4 ± 0A	2.6 ± 0.2A			
Leaf/ha (Kg)	1	1455.6 ± 24.4a	1143.5 ± 66.4ab	805.5 ± 25.7b	0.1438	0.4845	0.0322
	2	1016 ± 15ab	1553.5 ± 120.3a	1223 ± 23.2ab			
	Mean	1235.8 ± 39.7	1348.5 ± 75	1014.3 ± 41.2			
Stem/ha (Kg)	1	1964.1 ± 72.7	1080.1 ± 93.2	564.7 ± 38.8	<0.0001	<0.0001	0.5855
	2	3685.2 ± 191.1	1992.5 ± 24.5	1378.1± 88.1			
	Mean	2824.6 ± 176.9A	1536.3 ± 85.6B	971.4 ± 87B			
Dead Matter/ha (Kg)	1	323.9 ± 12	305.3 ± 28.7	167.5 ± 6.9	0.4263	0.1984	0.0966
	2	517.2 ± 6.7	167.4 ± 9.8	283.9 ± 11			
	Mean	420.6 ± 17.8	236.3 ± 18.4	225.7 ± 12.2			
Inflorescence/ha (Kg)	1	110.8 ± 7.8	17.2 ± 4	3.5 ± 0	0.0967	0.7532	0.8529
	2	39 ± 6.7	50 ± 1.9	0			
	Mean	74.9 ± 7.2	33.6 ± 3.3	0.14 ± 0.04			

a *variables = DM/ha, dry matter per hectare; L:S, leaf:stem ratio*.

b Different capital letters differ on the line for each variable; Different lowercase letters differ from each other in the treatment *period interaction for each variable analyzed.

The leaf:stem ratio (*P* = 0.0006) was greater in the Medium and Short treatments than in the Tall treatment. In contrast, the stem/ha variable (*P* < 0.0001) was greater in the Tall treatment, as shown in Table 2.

### Bromatological Composition of the Diet

The chemical composition of the diet did not differ among treatments (*P* > 0.05), with similar parameters, as shown in Table 3.

**Table 3:** Bromatological composition of the diet, based on the different structures of Capim Aruana (*Panicum maximum)*.

**Variables^a^ (% /kg de MS)**	**Treatments**			***P-*value**
	**Tall**	**Medium**	**Short**	
Mineral matter	9.8 ± 0.1	11.9 ± 0.2	10.7 ± 0.1	0.1068
NDF	66.5 ± 0.9	69.9 ± 0.7	65.6 ±0.3	0.3856
ADF	35.7 ± 0.3	36.7 ± 0.2	35.2 ± 0.6	0.7472
CP	15.9 ± 0.4	17.2 ± 0.4	17.9 ± 0.2	0.6599
EE	2.2 ± 0	2.5 ± 0	2.6 ± 0	0.0598
NIND	1.6 ± 0	2.3 ± 0	2.3 ± 0	0.4890
NIAD	0.30 ± 0	0.31 ± 0	0.30 ± 0	0.9748
LIGNIN	3.8 ± 0.1	4.6 ± 0	3.9 ± 0	0.2766

a *Variables = NDF, neutral detergent fiber; ADF, acid detergent fiber; CP, Crude Protein; EE, ethereal extract; NIND, nitrogen in neutral detergent; NIAD, nitrogen in acid detergent*.

## Discussion

### Ingestive Behavior

This experiment shows that young lamb grazing an upright tropical grass do not vary their GT due to the different sward heights, contrasting with what was discussed by Hodgson (9). This author, reviewing studies with temperate pastures, shows a linear increase in grazing time when the height or mass of the forage decreases. However, according to Sollenberger and Burns (28), this relationship in tropical pastures is not so consistent. Animals may graze taller or shorter tropical grasses for longer periods to compensate the limitations imposed either by pasture height, leaf size, number of stems or amount of herbage mass. However, rumination time in this study was longer in the Medium-height treatment, which demonstrates that factors other than structure can interfere in the GT of young animals grazing tropical pastures.

The leaf:stem ratio and the availability of green leaves in tropical pastures are key characteristics that affect animal ingestive behavior. For instance, Euclides et al. (29) studying *Panicum maximum* and *Brachiaria* spp. in southern Brazil showed that grazing time decreased with increments in the percentage of green leaves and leaf mass. Carvalho et al. (5) explains that the way leaves are presented to animals and how green leaves are apprehended, separately from stems and dead material, are important characteristics that should be considered in tropical pasture management in sheep production systems.

Despite the statistical difference in rumination time between treatments, it is important to consider that over 720 min of daily evaluation, rumination time in the Medium-height treatment was only 30 min greater than in the Short-height treatment. This small difference may then explain the lack of compensation observed for other activities in animals that exhibited shorter rumination times. In addition, these small differences in rumination time may not be physiological, since rumination activity is distributed throughout the day in periods ranging from 2 min to more than 1 h (30). Other activities were similar between treatments, which reinforces the idea that difference for rumination time may be associated with a natural variation in animal behavior that occurs throughout the day.

### Biting Rate

The greatest biting rates identified in this study occurred in animals exposed to the Short-height treatment (sward height of 25 cm). Biting rate and intake values by grazing animals are sensitive to variations in the mass and height of the pasture (31). In the case of an erect tropical grass, such as Capim Aruana, the shorter the pasture, the greater the biting rate. This response is consistent with observations in older lambs than those used in this study. Negri et al. (32) working with 120-day-old lambs grazing Capim Aruana (*Panicum maximum*) found that as sward height increased, animals spent more time (seconds) to achieve 20 mouthfuls. Stobbs (33) explains this behavior through the negative relationship between canopy height and density of the dry mass of green leaf blades, compromising the size of the bite due to increments in handling and chewing times. Similarly, Schwartz et al. (34) observed in sheep grazing pearl millet that at high pasture heights animals were forced to graze leaves individually due to leaf length, a behavior that decreased biting rate. Thus, there is a negative relationship between biting rate and height of tropical erect pastures (35). In fact, biting rate proved to be one of the variables that is most responsive to height variation of tropical erect grass.

A negative correlation was found between the rate of biting and the amount of inflorescence/ha in the pastures, whereas a positive correlation was detected between sward height and the presence of inflorescence. Likewise, Silva et al. (6) reported that the proportion of leaves in Aruana (*Panicum maximum*) and the presence of inflorescence influenced grazing strategy by lambs. This result shows the importance of avoiding the inflorescence in the pastures in order to facilitate lambs' grazing activities. Thus, the use of a mower or cattle grazing may contribute to manage pastures for grazing young lambs.

### Behavior Throughout the day

When analyzed over the hours of the day, the animals' ingestive behavior showed a natural behavioral pattern (36, 37). There was a peak of grazing in the early morning and late afternoon, and a moderate increase in rumination and idling activities during the late morning and early afternoon. Despite a decrease in GT by lambs during the hottest hours of the day (11:30 am−2:20 pm) the activity, although less frequent, was still observed in this study, contrary to results reported by Starling et al. (38) in tropical conditions. These authors evaluated Corriedale ewes poorly adapted to warm conditions and observed that the animals abruptly stopped their grazing activity at high environmental temperatures. The observation of grazing activity in the hottest hours of the day in our study may be related to climatic conditions that did not trigger high thermal stress and thus allowed grazing to occur at those times. This behavior demonstrates the need for ruminants to be constantly ingesting food (39) and shows that temperature may have a limited effect on the grazing behavior of animals in subtropical conditions.

The Short-height treatment showed that animals were already grazing more intensely than lambs in the other treatments during the early hours of the day. During the first hour of evaluation, animals in the Tall-height treatment grazed on average for 30 min and the Medium-height treatment for 10 min, whereas the Short-height treatment grazed on average for 40 min. Although there was not a significant difference in GT among treatments, the greater availability of leaves seems to prompt animals to graze earlier during the day.

In studies with sheep on bermudagrass pasture, Poli et al. (40) found that grazing was the activity that took up most of the lambs' time in the three production systems (lambs weaned on pasture; unweaned lambs exclusively on pasture; and unweaned lambs supplemented on pasture), consistent with results from the present study.

### Decision Tree Behavior

The Decision Tree analysis allowed us to explore the factors that influenced GT. The initial weight of the lambs had a key influence on GT. Lighter lambs grazed for longer periods than heavier lambs. The model highlights the importance of the structure of tropical pasture for animals under lower initial body weights, as height appears as the second factor influencing grazing time. In support of this, Emerenciano Neto et al. (41) report that among various structural characteristics, pasture height was a key variable influencing animals' foraging decisions.

Although there were no significant differences in GT between the different pasture-height treatments, important variability was observed for initial body weights within treatments and between years. Such variation allowed for distinguishing the effect of pasture height on initial body weight. The importance of animal size was also mentioned by Carvalho et al. (5). They explain that young and light lambs can be largely affected by herbage components, mainly due to the difficulty of bite formation by a small mouth area, which in turn influences the animals' grazing capacity.

The longer grazing times by lighter animals can lead to greater energy expenditures, with potential negative effects on performance. Thus, increasing body weights would be an ideal scenario for early weaned lambs entering tropical pastures. For larger lambs, the structure of tropical pasture has less influence on the time invested in grazing, whereas young lighter lambs may benefit from grazing shorter pastures, given that for animals of lower weight forage height had the greatest influence on GT.

These are innovative results that highlight the importance of initial weight for a weaned lamb to enter an erect tropical grass pasture. It is important to wean animals at proper body weights and developmental conditions, so that they can face the challenges imposed by tropical grasses, characterized by their large structure of leaves and stems combined with a fast growth rate.

These results also show the importance of assessing ingestive behavior as a tool to understand the factors that have direct effect on animal productivity. Our study shows important relationships between erect tropical grass pasture and young lamb size, generating innovative management decisions. In fact, there is a need to have a minimal lamb weight to face the challenges promoted by a tropical pasture. In this study the structure of a tropical pasture becomes less of a concern as the animal is heavier than 23 kg.

## Conclusions

Grazing time by young weaned lambs did not differ among different structures of an erect tropical grass sward, suggesting that other factors may influence foraging behavior. Biting rate proved to be the main variable that differed among the gradient of grazing structures presented. Body weight and height of the upright tropical grass pasture had a strong influence on lambs' ingestive behavior. Maximum pasture height and minimum body weight should be considered when young lambs graze an erect tropical grassland.

## Data Availability Statement

The raw data supporting the conclusions of this article will be made available by the authors, without undue reservation.

## Ethics Statement

The animal study was reviewed and approved by Universidade Federal do Rio Gande dos Sul Animal Care and Use Committee. Written informed consent was obtained from the owners for the participation of their animals in this study.

## Author Contributions

This study was developed by Animal Science Graduate Program at Universidade Federal do Rio Grande do Sul and it was financed by development agencies of the Brazilian Ministry of Education and Ministry of Science and Technology. Every author had important contributions on this manuscript. JS and LI developed the research project and fieldwork. CP is the head of the research project, being responsible for all parts of the study, from the project to the publication. JT was primarily responsible for the statistical analysis and results and discussion of the article. EM and JV contributed to the configuration of the manuscript and writing corrections. All authors contributed to the article and approved the submitted version.

## Conflict of Interest

The authors declare that the research was conducted in the absence of any commercial or financial relationships that could be construed as a potential conflict of interest.
